# Prevalence of Zika virus in blood donations: a systematic review and meta-analysis

**DOI:** 10.1186/s12879-019-4226-6

**Published:** 2019-07-06

**Authors:** Rongfei Liu, Xuanzhuo Wang, Yu Ma, Jianyong Wu, Chen Mao, Lihong Yuan, Jiahai Lu

**Affiliations:** 1 School of Public Health, Sun Yat-sen University, 74 Zhongshan Road 2, Guangzhou, 510080 Guangdong Province China; 2 Foshan Women and Children Hospital Affiliated to Southern Medical University, Foshan, Guangdong Province China; 3 One Health Center of Excellence for Research &Training, Sun Yat-sen University, Guangzhou, China; 4 Guangzhou Center for Disease Control and Prevention, Guangzhou, Guangdong Province China; 5 Department of Epidemiology, Guangdong Provincial Key Laboratory of Tropical Disease Research, School of Public Health, Southern Medical University, Guangzhou, China; 6 Key Laboratory of Tropical Disease Control of Ministry of Education, Sun Yat-sen University, Guangzhou, China; 7 State Key Laboratory of Vector-borne Infectious Disease (Hainan), Haikou, Hainan Province China

**Keywords:** Zika virus (ZIKV), Blood donors/donations, Blood screening tests

## Abstract

**Background:**

Transfusion-Transmitted Zika virus (TT-ZIKV) has become an emerging threat to world blood banks due to the fast spread of ZIKV epidemics and high rate of asymptomatic infections. For the risk assessment of ZIKV infection in blood products, relevant studies in blood donations or blood donors tested for ZIKV were collected and analyzed systematically. The overall prevalence of ZIKV infection were estimated through meta-analysis and potential risk factors were detected. The results will provide important clues for the protocol design of blood screening tests.

**Methods:**

Relevant articles about the rate of ZIKV detected in blood samples were identified from PubMed, Scopus and Web Of Science using key terms search strategy until October 7, 2017. Eligible articles were screened following inclusion and exclusion criteria. Meta-analysis and subgroup analyses were performed by software R3.4.1. Overall postdonation and posttransfusion follow-ups were analyzed.

**Results:**

Ten literatures (528,947 blood samples) were included for meta-analysis. The overall pooled prevalence of ZIKV (RNA and antibody) in blood donations was 1.02% (95%CI 0.36–1.99). The pooled prevalence of ZIKV RNA in blood donations was 0.85% (95%CI 0.21–1.88) less than the pooled prevalence of anti-ZIKV antibodies 1.61% (95%CI 0.03–5.21), however the difference was not statistically significant (*p* = 0.52). The prevalence varied significantly in different geographical regions (*p* < 0.001). Blood donations were more than two times likely to be infected by ZIKV in Zika epidemic period (1.37, 95%CI 0.91–1.91) than in non-epidemic period (0.61, 95%CI 0–2.55). The prevalence of anti-ZIKV antibodies (1.61, 95%CI 0.03–5.21) was almost twice as much as ZIKV nucleic acid detected in blood donations (0.85, 95%CI 0.21–1.88). However, statistically significant differences were not observed. A total of 122 ZIKV positive blood donors were followed, of which 48 (39%) reported symptoms postdonation, but none of the 13 followed recipients reported any clinical symptoms related to Zika infection posttransfusion.

**Conclusion:**

The pooled prevalence of Zika infection in blood donations was 1.02%. The prevalence varied greatly and reached to high-risk level in most of the situations. The results suggest that nucleic acid tests (NAT) for blood screening and pathogen reduction/inactivation technology (PRT) should be implemented in Zika-endemic areas and appropriate strategies should be designed according to different conditions. More studies are needed in the future to provide more evidence.

**Electronic supplementary material:**

The online version of this article (10.1186/s12879-019-4226-6) contains supplementary material, which is available to authorized users.

## Background

As a zoonotic pathogens and reemerging arbovirus, Zika virus (ZIKV) has caught extensive attention since its outbreak in Brazil 2015 [1]. ZIKV belongs to the genus Flavivirus, family Flaviviridae. It shares many biological and molecular characteristics in symptoms, genome and pathogenicity with other family members such as dengue virus (DENV), yellow fever virus (YFV), West Nile virus (WNV) and Japanese encephalitis virus (JEV) [2]. Approximately 80% of Zika infections are asymptomatic [3, 4], the remainder usually develop non-specific clinical symptoms such as mild fever, rash, arthralgia, myalgia, conjunctivitis and headache [5]. Nevertheless, sometimes ZIKV infection can lead to much severer neurological complications, such as Guillain-Barre Syndrome in adults [6, 7] and microcephaly in neonates [8].

ZIKV is transmitted to humans mainly through Aedes mosquitoes bite, but it can also be spread by sexual activities, maternal-fetal pathway, physical contact and blood transfusion [3, 9]. ZIKV RNA has been detected in blood, semen, saliva, urine and other biofluids samples, but the viremia period of ZIKV is not definite. It was estimated that the median incubation period for the infection was 5.9 days and the virus was detectable in blood for 9.9 days [10]. Notably, ZIKV RNA can persist in serum up to 54 days in some cases [11]. So, asymptomatic infectors in viremia period can be a nonnegligible source of ZIKV transmission and largely increase the risk that blood donations were already contaminated before collection.

WHO [12] and FDA [13] have instituted multiple interventions to reduce the risk of transfusion-transmission (TT) ZIKV, such as deferral of blood collection in endemic areas and donors returned from Zika-endemic countries within 28 days; import of blood from low-risk regions; screening blood donations by ZIKV NAT (Nucleic Acid Tests) and/or treating them with PRT (Pathogen Reduction/inactivation Technology). On March 30, 2016, the first ZIKV NAT assay for blood screening was permitted by FDA for emergency use, enabling the blood collection in Puerto Rico resumed [14]. However, there are opposite attitudes towards mandating routine screening of ZIKV in blood collections because it is time-consuming and costly. Some consider that the TT risk of Zika in their country is low [15, 16], and there is no urgent need to introduce universal screening of donated blood for ZIKV [17]. On the other hand, some studies mentioned that present measures were not effective enough to prevent transfusion of ZIKV RNA-reactive blood products, and ZIKV NAT should be used [18, 19]. Their attitudes varied greatly depend on the prevalence rate of ZIKV considered as low or high by different public health officials in different areas, but the cut-off value hasn’t been revealed. ZIKV TT risk modeling is not yet available to date due to the absence of critical parameters [20] and US FDA Zika guidance remained to be evaluated by a formal risk assessment and stakeholder consultation [21]. Many factors can influence the interventions to reducing TT risk of ZIKV, including social, economic and ethical factors, but the foremost factor is the infection rate of ZIKV in blood components, which should be a topic for further discussions. In this study, we performed the first systematic review and meta-analysis to assess the prevalence of ZIKV in blood donors or blood donations and find out potential risk factors. The result will provide valuable reference information for strategy making to ensure blood supply safety.

## Methods

### Search strategy

This systematic review was performed in accordance to the PRISMA guidelines [22] and registered in PROSPERO (registration number: CRD42018088046). Medical literature databases search was conducted in PubMed, Scopus and Web Of Science using key terms: (“ZIKV” OR “zika virus” OR “arboviruses”) AND (“blood” OR “seroprevalence” OR “transfusion” OR “serum” OR “plasma” OR “serologic” OR “serological”) restrict to [Title] and published before October 7, 2017. Relevant papers were also identified from the reference lists of previous publications. All literatures were imported to NoteExpress software (V3.0) after initial search, and duplications were removed.

### Study selection

The selection process was conducted by two researchers (RFL and XZW) independently in 2 steps. First, we browsed through titles and abstracts of each study, any type of study such as cross-sectional/prevalence study, cohort study, case-control study and screening study were eligible for next inspection as long as the survey of ZIKV or arboviruses in human blood samples was mentioned in the study. Second, we went full-text screening and included studies that focused on ZIKV infection in blood donors or donations. The detection of ZIKV was confirmed by molecular or serological methods with RNA and/or IgM/IgG detected in samples. Studies were excluded when: 1). the types of articles are overviews, abstracts, case-reports and comments. 2). the articles are not in English; 3) the purpose of the study is to evaluate detection methods of ZIKV; 4) the samples size is less than 500. Any disagreement between reviewers was resolved by discussion with a third experienced investigator (YM).

### Data extraction and quality assessment

All data from included studies were extracted independently by 2 reviewers (RFL and XZW) using the same Microsoft Excel spreadsheet and any discrepancies were resolved by consensus. The following data were recorded: Author (study year), sample source, study period (*epidemic means the time is during local Zika-epidemic), study area, number of ZIKV infected samples (Event), total number of samples tested (Number), virus loads (if any), and detection method. As studies finally included were all prevalence-study, so, the quality score of studies was assessed by an 11-item checklist recommended for cross-sectional/prevalence study by the Agency for Healthcare Research and Quality (AHRQ) [23]. An item would be scored “0” if it was answered “NO” or “UNCLEAR”; scored “1” for “YES”. Article quality was classified as follows: low quality = 0–3; moderate quality = 4–7; high quality = 8–11.

### Statistical analysis

The proportion of samples infected with ZIKV in each study was combined to give a pooled prevalence of ZIKV in blood donations. All analyses were performed with R 3.4.1 software (https://www.r-project.org). Heterogeneity was assessed using the *I*^*2*^ statistic and Chi-square test (*I*^*2*^ value of 25, 50, and 75% indicates low, moderate, high and very high heterogeneity [24], and *P* value < 0.10 define a statistically significant degree). Random effects model was used for high level of heterogeneity. The data were converted using Freeman-Tukey Double arcsine transformation to satisfy the normal distribution. Subgroup analyses were conducted according to study period, study area and detection method, difference of subgroups was tested by Cochran Q statistic, the significant level (two-tailed) was set at *P* value < 0.05. Sensitivity analyses were performed to evaluate the stability of analyses, and potential publication bias was assessed by funnel plots and Egger’s test.

## Results

### Study selection

**Fig. 1:**
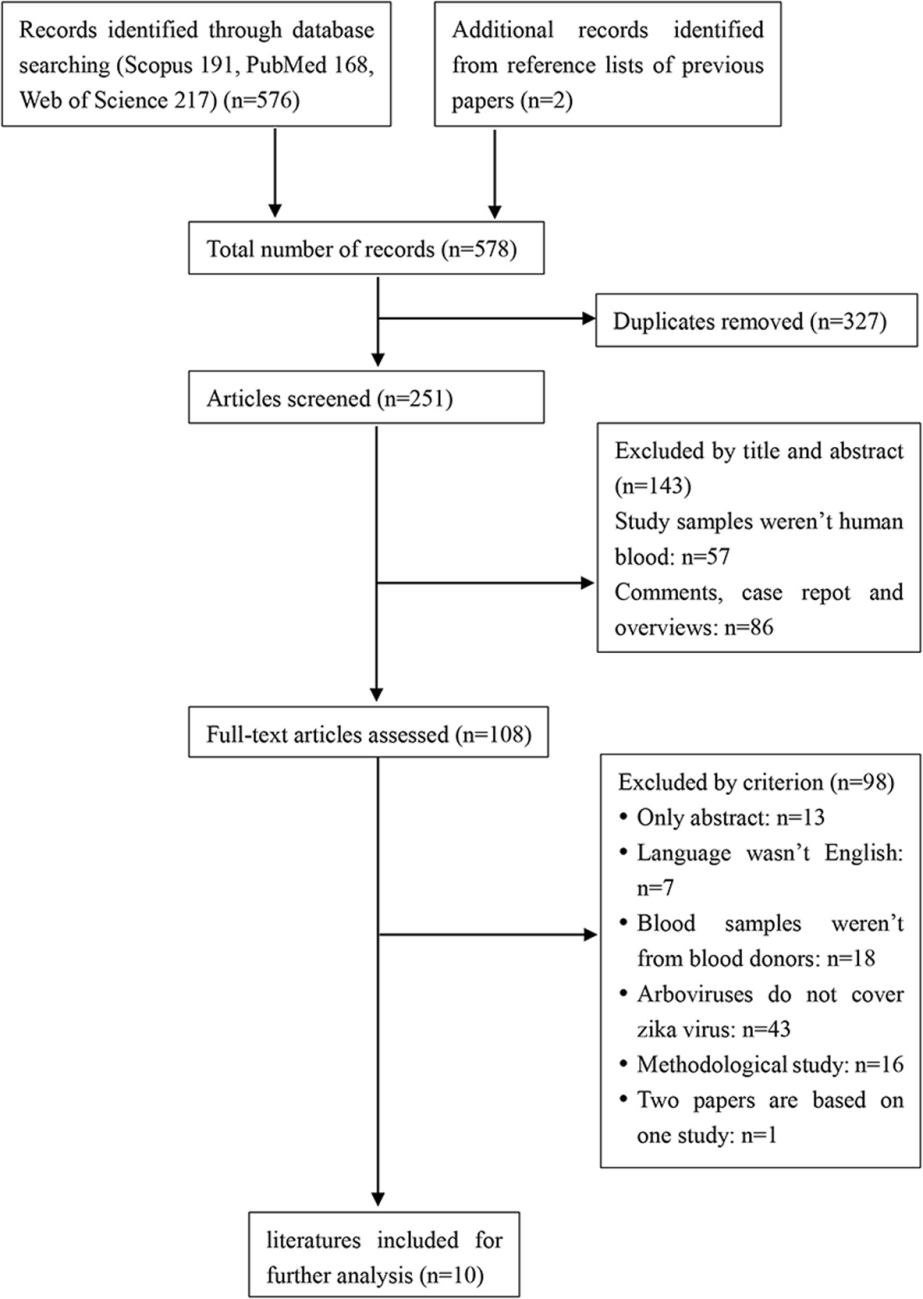
Flow diagram of assessment of studies identified in the meta-analysis

The search strategy identified 578 citations at the initial search, of which 327 were removed as duplicates. After screening process, 10 literatures were included (Fig. 1). Agreement between reviewers for assessment of study eligibility was 100%. Detailed characteristics and quality score of all included studies are provided in Table 1.

**Table 1:** Characteristics and quality assessment score of 10 included literatures

Study	Author study year	Sample source	Study period (^a^epidemic)	Area	Event	Number	Detection method	Viral loads	Quality score
p1	Bierlaire2014	blood collections tested prospectively and retrospectively	3 months^a^	French Polynesia	42	1505	lab developed NAT assay	2.5e3–8.1e6 copies/ ml	8
p2	Chevalier2016	blood donation screening in 2 largest blood centers	4 months^a^	Puerto Rico	190	21,468	Cobas Zika ID-NAT	NM	7
p3	Gake2015	blood donors from six sites representing eco-environments	2 months	Cameroon	53	1084	Euroimmun anti-Zika NS1 IgG ELISA	NM	4
p4	Williamson2016	blood donor screening at laboratories	2 months	several states of United State	5	466,834	Procleix ZIKV assay, RT-PCR, CDC IgM/IgG-ELISA	10^2^ IU	7
p5	Borena2016	blood donors at randomly selected donation sites	3 months	Austria	4	1001	Euroimmun anti-Zika NS1 IgM/IgG ELISA	NM	6
p5.1					0	1001	RealStar zika virus RT-PCR kit 1.0		
p6	Gallian2016	consecutive blood donations	5 months^a^	Martinique	76	4129	RealStar Zika virus RT-PCR Kit_1.1	2.1–6.5log10 copies/ml	6
p7	Slavov2016	volunteer blood donors	7 months^a^	Brazil	37	1393	In-house method RNA test	mean 7714 copies/ml	8
p8	Kuehnert2016	screened blood donations	2 months^a^	Puerto Rico	68	12,777	Cobas Zika NAT assay	NM	4
p9	Adams2016	screened blood donations	3 months^a^	Puerto Rico	143	18,163	Cobas Zika NAT assay	NM	4
p10	Aubry2013	volunteer blood donors	27 months	French Polynesia	5	593	self-developed indirect IgG ELISA	NM	5

Note: ^a^epidemic: the study period is during Zika epidemic period

*NM* not mentioned

Of the 10 included literatures (p1[19], p2 [25], p3 [26], p4 [27], p5 [17], p6 [28], p7 [29], p8 [30], p9 [31], p10 [32]), a large majority were performed in the year of 2016 (7 studies, p2,p4,p5,p6,p7,p8,p9) and each has one in 2013 (p10),2014 (p1) and 2015 (p3). Puerto Rico is the most interested location with 3 studies (p2,p8,p9) conducted here, followed by French Polynesia with 2 studies (p1,p10). Study areas were classified by geographical continents for the sake of analysis: North America (Puerto Rico, United States):4 studies, South America (Martinique, Brazil):2 studies, Oceania (French Polynesia):2 studies, Europe (Austria):1study, and Africa (Cameroon):1 study. As to the detection methods, 7 studies detected ZIKV RNA and 2 studies detected anti-ZIKV antibodies. One research (p5) have used both nucleic acid test and antibody test in all samples separately and did not give a conclusion that weather it’s false positive/negative or not, so we took it as two studies (p5, p5.1) in future meta-analysis. Methodological quality assessment showed 2 studies were of high quality (score 8) and 8 studies were of moderate quality (mean score 5.4) (Table 1).

### Overall prevalence of Zika virus in blood donations

**Fig. 2:**
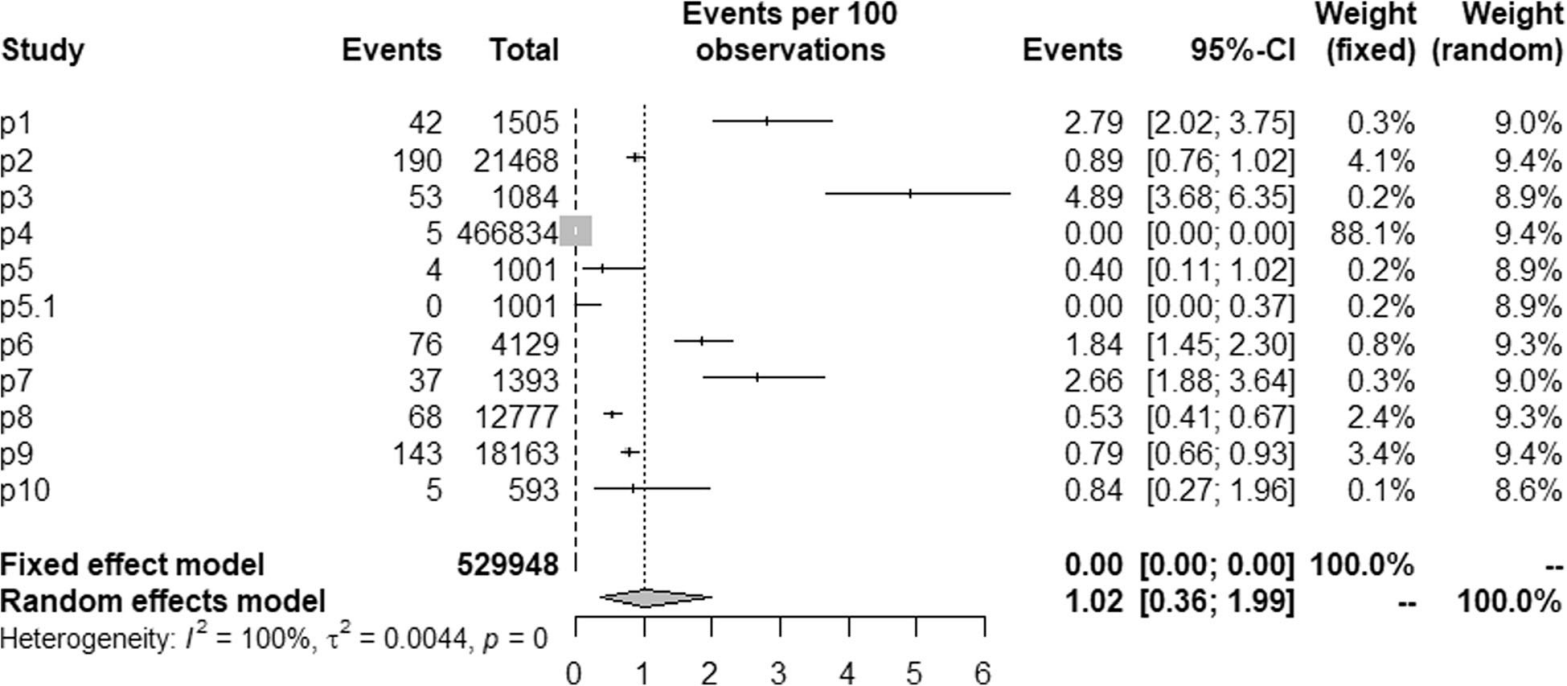
Forest plot of the pooled prevalence of ZIKV in blood donations

A total of 528,947 blood samples were tested in 10 literatures, the prevalence of Zika virus in blood donations range from 0 to 4.89%, and ZIKV RNA detection rate range from 0 to 2.79%, ZIKV antibody seroprevalence range from 0.40 to 4.89%. All studies have samples more than 500 and 4 of which more than 10,000 (p2, p4, p8, p9). A very high level of heterogeneity was observed (*I*^*2*^ = 99.5%, *p* < 0.001), so, random effect model was used to estimate the overall pooled prevalence of ZIKV (RNA and antibody) in blood donations as 1.02% (95%CI 0.36–1.99) (Fig. 2).

### Prevalence of Zika virus in blood donations in different conditions

As to the study period, most of the studies (7/10) were performed in 2016 and only one study in other years, so it would be hard to deduce the prevalence trend of ZIKV in recent years. But since the prevalence of Zika virus in blood is presumably influenced by local endemic situation, we divided the studies into 2 parts according to whether the study was performed during local epidemic/outbreak or not. Epidemic and outbreak situation is defined as a series of autochthonous infection cases were reported in a short time and epidemiological correlation between cases were observed. Non-epidemic/outbreak situation is identified in those studies: study conducted in areas where ZIKV infection cases have been reported long ago but no exceptionally increases occurred in study time (p3); study performed in outside of active-transmission areas (p4); study in risk area but no cases have been reported (p5); and study collected samples before ZIKV emergence (p10). Six studies (p1,p2,p6,p7,p8,p9) were performed when Zika is circulating and the pooled prevalence of Zika virus in blood donations was 1.37% (95%CI 0.91–1.91). Five studies (p3,p4,p5,p5.1,p10) were performed when non Zika-epidemics and the pooled prevalence was 0.61% (95%CI 0–2.55) (Additional file 1: Figure S1). The prevalence in epidemic areas was more than two-times higher than non-epidemic areas, but it was not statistically significant (*p* = 0.39).

Stratified by geographical continents, Africa, Europe, Oceania and South America have too few studies (less than 3), so the pooled prevalence rate in those areas may not be representative. North America has the largest number of studies: 4, and the pooled prevalence was 0.42% (95%CI 0–1.49, Additional file 2: Figure S2). The rate in different continents varied considerably (*p* < 0.001). Unfortunately, no study is available in Asia.

Stratified by detection method, the pooled prevalence of ZIKV RNA in blood donations was 0.85% (95%CI 0.21–1.88) less than the pooled prevalence of anti-ZIKV antibodies 1.61% (95%CI 0.03–5.21, Additional file 3: Figure S3), however the difference was not statistically significant (*p* = 0.52).

Significant heterogeneity was observed for pooled analysis of prevalence rate in overall included studies and subgroup studies (*p* < 0.001). The heterogeneity was considered very high with *I*^*2*^ > 95% in all pooled analyses and did not change considerably in different subgroups. Sensitivity analysis showed that the pooled rate was quite stable when excluding any of the studies. Funnel plot and Egger’s test indicated the existence of publication bias (Additional file 4: Figure S4, *p* = 0.003).

### Results of follow-ups

In all included studies, a total of 122 ZIKV reactive blood donors were followed in three studies (p1:42, p4:5, p6:75), of which 48(39%) reported symptoms within 1–10 days of postdonation, and 3 cases (p4) were retrospectively confirmed had shown symptoms before donation. Two studies (p1, p4) have traced down to the recipients to whom ZIKV reactive blood donations were transfused. Overall, among the 13 recipients (p1:12, p4:1) investigated, 3 of them have developed anti-ZIKV IgG antibodies after received RBCs (p1). None of recipients reported any clinical symptoms consistent with ZIKV infection within 2 months of posttransfusion.

## Discussion

Flavivirus transmission through blood transfusion has been recorded for Dengue virus [33], West Nile virus [34], Yellow Fever vaccine virus [35] and Zika virus [9, 36]. The major challenges in preventing transfusion-associated transmission of ZIKV are the high rate of asymptomatic infections and the high proportion of infected people in endemic-areas [37]. Asymptomatic infectors have long been a challenging aspect in the control and prevention of infectious disease, for their ability to spread virus and high mobility without surveillance. Asymptomatic blood donors during viremia period can pose a great threat to blood supply system with viremia of Zika virus reaching to a high level exceeding 10^8^copies/mL [27]. Therefore, an extensive investigation of ZIKV prevalence in blood donations is quite essential to provide information for interventions implemented to ensure the safety of blood transfusion.

Through this meta-analysis, we concluded the overall prevalence of ZIKV in blood donations was 1.02%, which is higher than DENV RNA (around 0.19% [38]~ 0.4% [39]) and CHIKV RNA (0.36–0.42% [40]) detected in blood donors, and much higher than the rates of window period infections detected by NAT screening for HIV, HCV and HBV over the past two decades [41]. AABB (formerly the American Association of Blood Banks) have categorized dengue as high priority agents in terms of threats to US transfusion recipient safety [42]. Zika virus should also be classified as high-risk agent according to the criteria and even severer than dengue for its possible permanent neurological damage. Routine nucleic acid testing for WNV was performed for all US donations in 2003 and prevented thousands of WNV transfusion-transmissions, more than 27 million donations were tested during transmission periods with 1576 WNV RNA-positive donations identified [43], which is much lower than the rate of ZIKV prevalence in blood donations we summarized here. The virus load of ZIKV in blood donations in included studies ranged from 10^2^~10^7^ copies/ml, even though the dose required to cause infection in a recipient is unknown due to few recipients were investigated and the difficulty to confirm a TT case excluding vectorial transmission, the virus load is much higher than WNV screened in blood donations [43]. Thus, since routine blood screening test was already implemented for WNV, ZIKV should also be taken into consideration, especially in ZIKV-endemic areas.

The prevalence of anti-ZIKV antibodies (1.61%) in blood donation was almost twice as much as ZIKV RNA prevalence (0.85%), this may due to antibodies can persist longer in blood than nucleic acid, thus are more likely to be detected. But the existence of antibodies doesn’t mean the blood is contagious, for IgG antibody only indicate remote infections while IgM represent recent infections. On the contrary, a mere absence of ZIKV nucleic acid in blood samples does not absolutely rule out the existence of virus. So, both ZIKV RNA and antibodies in blood donations implied risks in blood supply system. Here we calculated the overall pooled prevalence of ZIKV including both ZIKV RNA and antibody, and then computed separately within each method. The 3 included antibodies-detected studies all tested for anti-ZIKV IgG antibodies, only 1 study (p5) tested both IgM and IgG and merely 3 cases were reactive with IgM in all included samples. Additionally, antibody detection assay may be compromised by cross-reacting antibodies and lead to false-positive results [44]. So, antibody-test may not be an appropriate screening assay for blood inspection. But there was no statistically significant difference between two detection methods, which may be due to the limited amount of studies in antibody detection. The prevalence of ZIKV in blood donations during epidemic period (1.37%) was more than two times higher than in non-epidemic period (0.61%). Blood donations collected during non-epidemic period were likely get infected because of the donors may have travel history to ZIKV endemic areas, and get infected without notice before donation. Another possibility that ZIKV has already transmitted into the study areas where are outside of the active-transmission regions or at risk of virus invading. There are four studies conducted in non-epidemic period, and three of them were detected ZIKV antibodies. So, it’s also a possibility that false-positive results were made because of antibody cross-reacting with other flavivirus. The result shows that South America has the highest prevalence of ZIKV in blood donations (2.17%), which is consistent with the geographic distribution of Zika infection [45, 46] and Brazil contributed most of the cases. Africa has a high prevalence of anti-Zika IgG (4.89%), which can be attributed to the sporadic circulation history of Zika in Africa [45].

Because of the high volume of blood needed and difficulties in blood transportation to remote areas, as well as quickly-spread and large-scale of Zika virus epidemics, it is not practical to import blood from low-risk regions to supply most endemic areas. So, safe and cost-effective technology was urgently needed to guard blood supply safety. The existing blood donor-screening ZIKV NAT assays demonstrate excellent sensitivity [47] and effectiveness. The risk of ZIKV TT infection can be reduced greatly of 29% by antibody screening and 7% by symptom-based screening [10]. Another important measure to ensure blood safety is PRT, but the licensed methods are only available for plasma and platelets now. Fortunately, many studies have shown that amustaline/glutathione treatment for red blood cells can effectively inactivate Zika [48], Dengue [49] and Chikungunya virus [50], and other methods aimed at red blood cells were under clinical trials and promising to be applied in the future [51]. Cost of screening and PRT assays is another crucial concern before carrying out a policy. Although the screening assays are high-cost, the adoption of a new system could be effective for many viruses and thus eliminating other existing tests along with their costs, so that the total costs would not be increased greatly. Blood donations NAT screening and PRT were strongly suggested in Zika virus-endemic areas. Given 39% of asymptomatic donors showed symptoms postdonation, it will be helpful to develop follow-ups after blood collection. Areas with no capacity to provide screening assays to all blood donations and non-endemic areas should strengthen general inspection measures, such as detailed questionnaire survey before donation and screening tests should focus on at-risk recipients, such as pregnant women.

There are spaces for improvements to this review: Firstly, the amount of eligible studies was not enough for deep analysis of subgroups, for some subgroups contained only 1 study and some are null, which could reduce the accuracy of results. Secondly, significant high heterogeneity and publication bias were observed. Several factors might contribute to the existence of heterogeneity, for example, socioeconomic status, sanitary conditions and most importantly, the method used to detect ZIKV infection and the diagnostic cutoffs adopted. In addition, the heterogeneity of single-rate meta-analysis is usually higher than two-group study, such as case-control study and RCTs, because the data were extracted only from one group. It is common to see that *I*^*2*^ statistic often exceed 90% in some other meta-analysis studies [52, 53]. So *I*^*2*^ statistic should not be the only standard to judge the reliability of a study. Based on the precisely sampling methods and detailed subgroup analyses, we believe that the pooled prevalence of ZIKV in blood donations calculated in this study are of high reference value. Thirdly, those studies that detected antibodies didn’t confirm the result with RNA tests, this caused much uncertainty in the interpretation of the result. Finally, it would be better if detailed information was provided in included studies, such as the demographic characteristics of blood donors or the types of blood products (plasma, platelet, red cell and whole blood). With attention on ZIKV increases, more research data are expected in the future to provide more insights for intensive research.

## Conclusions

Based on this meta-analysis, the prevalence of Zika infection in blood donations is at high-risk level of 1.02%, the prevalence varied greatly in different areas. NAT blood screening tests and PRT are recommended in Zika virus-endemic regions and appropriate strategies should be made according to different conditions. More studies are needed in future to carry out more detailed researches.

## Additional files

Additional file 1: **Figure S1.** Forest plot of the prevalence of ZIKV in blood donations in different epidemic situations of Zika. (DOCX 71 kb)

Additional file 2: **Figure S2.** Forest plot of the prevalence of ZIKV in blood donations in different areas. (DOCX 84 kb)

Additional file 3: **Figure S3.** Forest plot of the prevalence of ZIKV in blood donations detected by different methods. (DOCX 70 kb)

Additional file 4: **Figure S4.** Funnel plot of the included studies in meta-analysis. (DOCX 30 kb)

## Data Availability

All data generated or analysed during this study are included in this published article and its supplementary information files.

## Abbreviations

NAT: Nucleic Acid Test
PRT: Pathogen Reduction/inactivation Technology
TT: Transfusion-Transmitted/Transmission
ZIKV: Zika virus
