# Supplementary material for: Prevalence of Zika virus in blood donations: a systematic review and meta-analysis
Source: BMC Infect Dis. 2019 Jul 6;19:590. doi: 10.1186/s12879-019-4226-6 (PMC6612077; doi:10.1186/s12879-019-4226-6)
Supplement: Supplementary file 2 [file 12879_2019_4226_MOESM2_ESM.docx]

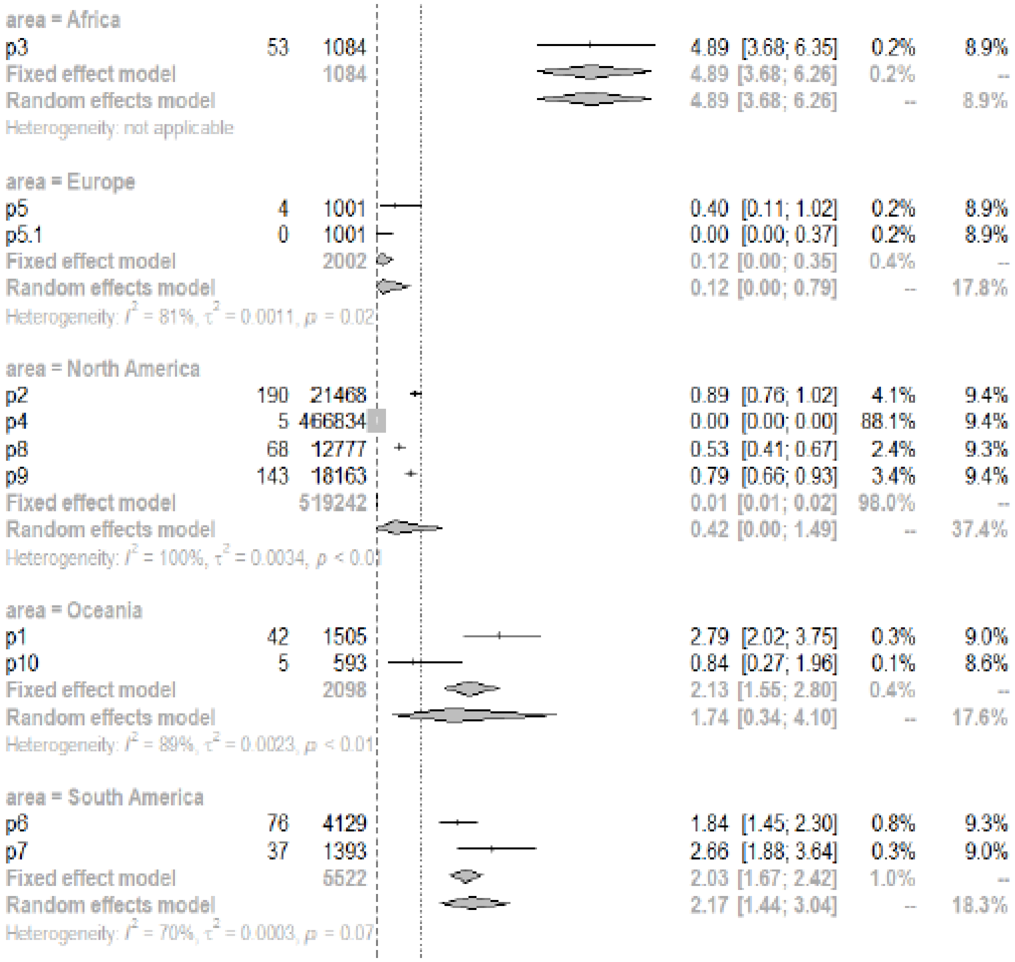

Figure S 2 Forest plot of the prevalence of ZIKV in blood donations in different areas
