# Supplementary material for: Prevalence of Zika virus in blood donations: a systematic review and meta-analysis
Source: BMC Infect Dis. 2019 Jul 6;19:590. doi: 10.1186/s12879-019-4226-6 (PMC6612077; doi:10.1186/s12879-019-4226-6)
Supplement: Supplementary file 3 [file 12879_2019_4226_MOESM3_ESM.docx]

Study	Events	Total	Events	95%-CI	Weight (fixed)	Weight (random)
method = Antibody test						
p3	53	1084	4.89	[3.68; 6.35]	0.2%	8.9%
p5	4	1001	0.40	[0.11; 1.02]	0.2%	8.9%
p10	5	593	0.84	[0.27; 1.96]	0.1%	8.6%
Fixed effect model		2678	1.81	[1.33; 2.35]	0.5%	--
Random effects model			1.61	[0.03; 5.21]	--	26.4%
Heterogeneity: $I^2 = 96\%$, $\tau^2 = 0.0078$, $p < 0.01$						
method = RNA test						
p1	42	1505	2.79	[2.02; 3.75]	0.3%	9.0%
p2	190	21468	0.89	[0.76; 1.02]	4.1%	9.4%
p4	5	466834	0.00	[0.00; 0.00]	88.1%	9.4%
p5.1	0	1001	0.00	[0.00; 0.37]	0.2%	8.9%
p6	76	4129	1.84	[1.45; 2.30]	0.8%	9.3%
p7	37	1393	2.66	[1.88; 3.64]	0.3%	9.0%
p8	68	12777	0.53	[0.41; 0.67]	2.4%	9.3%
p9	143	18163	0.79	[0.66; 0.93]	3.4%	9.4%
Fixed effect model		527270	0.01	[0.00; 0.01]	99.5%	--
Random effects model			0.85	[0.21; 1.88]	--	73.6%
Heterogeneity: $I^2 = 100\%$, $\tau^2 = 0.0042$, $p = 0$						
**Fixed effect model**		**529948**	**0.00**	**[0.00; 0.00]**	**100.0%**	**--**
**Random effects model**			**1.02**	**[0.36; 1.99]**	**--**	**100.0%**
Heterogeneity: $I^2 = 100\%$, $\tau^2 = 0.0044$, $p = 0$						

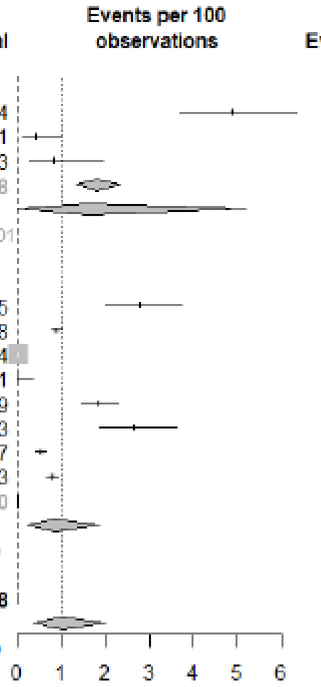

Figure S 3 Forest plot of the prevalence of ZIKV in blood donations detected by different methods
