# Supplementary material for: Prevalence of Zika virus in blood donations: a systematic review and meta-analysis
Source: BMC Infect Dis. 2019 Jul 6;19:590. doi: 10.1186/s12879-019-4226-6 (PMC6612077; doi:10.1186/s12879-019-4226-6)
Supplement: Supplementary file 4 [file 12879_2019_4226_MOESM4_ESM.docx]

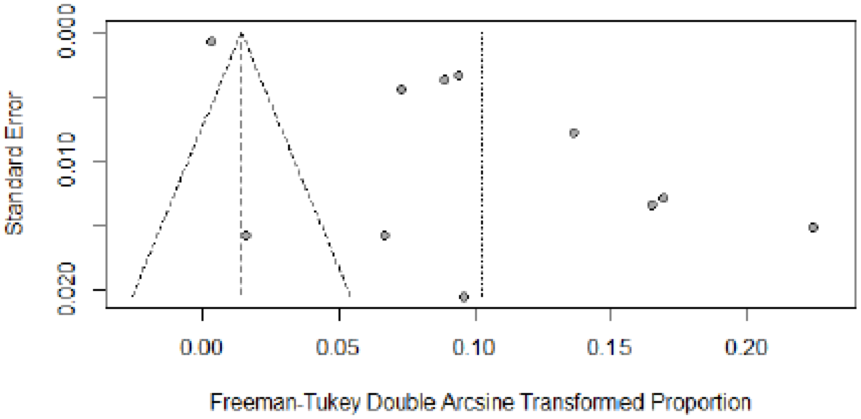

Figure S 4 Funnel plot of the included studies in meta-analysis
